# Novel Gastroretentive Floating Pulsatile Drug Delivery System Produced via Hot-Melt Extrusion and Fused Deposition Modeling 3D Printing

**DOI:** 10.3390/pharmaceutics12010052

**Published:** 2020-01-08

**Authors:** Nagi Reddy Dumpa, Suresh Bandari, Michael A. Repka

**Affiliations:** 1 Department of Pharmaceutics and Drug Delivery, School of Pharmacy, The University of Mississippi, Oxford, MS 38677, USA; ndumpa@go.olemiss.edu (N.R.D.); sbandari@olemiss.edu (S.B.); 2 Pii Center for Pharmaceutical Innovation & Instruction, The University of Mississippi, Oxford, MS 38677, USA

**Keywords:** hot-melt extrusion, fused deposition modeling, 3D printing, floating systems, pulsatile release, chronotherapeutic delivery

## Abstract

This study was performed to develop novel core-shell gastroretentive floating pulsatile drug delivery systems using a hot-melt extrusion-paired fused deposition modeling (FDM) 3D printing and direct compression method. Hydroxypropyl cellulose (HPC) and ethyl cellulose (EC)-based filaments were fabricated using hot-melt extrusion technology and were utilized as feedstock material for printing shells in FDM 3D printing. The directly compressed theophylline tablet was used as the core. The tablet shell to form pulsatile floating dosage forms with different geometries (shell thickness: 0.8, 1.2, 1.6, and 2.0 mm; wall thickness: 0, 0.8, and 1.6 mm; and % infill density: 50, 75, and 100) were designed, printed, and evaluated. All core-shell tablets floated without any lag time and exhibited good floating behavior throughout the dissolution study. The lag time for the pulsatile release of the drug was 30 min to 6 h. The proportion of ethyl cellulose in the filament composition had a significant (*p* < 0.05) effect on the lag time. The formulation (2 mm shell thickness, 1.6 mm wall thickness, 100% infill density, 0.5% EC) with the desired lag time of 6 h was selected as an optimized formulation. Thus, FDM 3D printing is a potential technique for the development of complex customized drug delivery systems for personalized pharmacotherapy.

## 1. Introduction

Maintaining a constant plasma drug concentration is not beneficial in all disease conditions. Some diseases may require pulse delivery of drugs to avoid unwanted adverse effects and drug exposure [1]. Certain diseases, such as bronchial asthma, angina pectoris, ulcers, and rheumatoid arthritis, are regulated by the circadian rhythm of the body and require drug administration at specific times of a day, particularly in the early morning hours. Such diseases require pulsatile drug delivery after a lag time to improve patient compliance and drug adherence. Pulsatile drug delivery systems (PDDS) provide a timely pharmacological effect to the patient, while preventing unwanted sustained drug exposure. PDDS does not interrupt patients normal sleep patterns following an evening dose of medication. Moreover, pulsatile systems can prevent detrimental drug–drug interactions without changing the administration schedule of patients taking multiple medications at the same time and can enhance patient compliance [2,3,4]. To address these issues, a drug delivery system that delivers a pulsatile release of drugs after a pre-determined lag time is necessary. Thus, drugs administered at bedtime would be released in the early morning hours and be available to alleviate the symptoms and improve the quality of life of the patients.

Various biological factors influence the transit time of drugs in the upper gastrointestinal tract and are a challenge to drugs that are locally active in the stomach, unstable at a high pH, or that have poor solubility in the lower parts of the gastrointestinal tract [5,6,7]. A strategy to increase the residence time of the drugs to overcome the above-mentioned drawbacks is necessary for optimal therapeutic outcomes. Numerous strategies have been used to increase the residence time of the dosage forms in the stomach, including mucoadhesive systems [8,9], high-density systems that sink to the bottom of the stomach [10], swelling systems [11] and floating systems [12,13,14]. Among these strategies, floating systems are considered superior as they do not interfere with the physiological activity of the gastrointestinal tract [15]. Floating systems are further subdivided into effervescent and non-effervescent floating systems. The effervescent systems use gas-forming agents, whereas the non-effervescent systems are formulated with gel-forming polymers or hollow microspheres [16]. 

Hot-melt extrusion (HME) is a well-known technique used in the plastic, rubber, and food industries. Over the last three decades, its use in drug delivery and research has tremendously increased owing to the advantages associated with this technology, such as high efficiency, solvent-free, innovative applications, and continuous manufacturing [17]. Although HME is most widely used in the preparation of amorphous solid dispersions, it is also used in the development of many innovative applications, such as taste masking, abuse-deterrent formulations, chronotherapeutic systems, topical formulations, semi-solid dosage forms, and co-amorphous systems [18,19,20,21,22]. Recently, the interest of researchers has shifted to additive manufacturing of pharmaceutical dosage forms where HME is combined with three-dimensional (3D) printing.

Three-dimensional printing is a process in which digitally controlled 3D objects are produced by the deposition of materials in a layer-by-layer manner [23]. Previously it was widely used in automobile, robotics, aerospace, and other industries for rapid prototyping purposes. Because of the widespread availability of commercial 3D printers and the recent FDA approval of the first 3D printed dosage form (Spritam), its use in pharmaceutical research has greatly increased [24]. The main reason for the increased attention of researchers on 3D printing is because of its potential to create complex, customized, and personalized-on-demand dosage forms. Recently, fused deposition modeling (FDM) 3D printing has been used in the preparation of various novel drug delivery systems, such as personalized vaginal progesterone rings [25], channeled tablets for superior disintegration and dissolution [26], personalized oral delivery devices [27], and topical nose-shaped device for the treatment of acne [28].

In this study, HME technology-paired FDM 3D printing and conventional direct compression methods were utilized to produce novel core-shell floating pulsatile tablets with a predetermined lag time of 6 h. Asthma is a chronic inflammatory condition of the airways and symptoms of this condition include shortness of breath, chest tightness and coughing, which are worsened at early morning hours due to regulation of the circadian rhythm. So, theophylline, which is widely used for the symptomatic treatment of chronic asthma, was chosen as a model drug. However, the in vivo performance of the developed drug delivery system will be investigated in our future studies. The aim of the developed drug delivery system is to achieve both floating characteristics and pulsatile release after the desired lag time to improve therapy and quality of life of patients with asthma.

## 2. Materials and Methods 

### 2.1. Materials

Theophylline anhydrous (≥99% pure) was purchased from Sigma-Aldrich (St. Louis, MO, USA). Hydroxypropyl cellulose (HPC, Klucel LF) and ethyl cellulose (EC, Aqualon EC N14) were donated by Ashland Inc. (Covington, KY, USA). Croscarmellose sodium (Ac-Di-Sol^®^) and microcrystalline cellulose (Avicel PH^®^ 102) were provided by FMC Biopolymer (Newark, DE, USA). Magnesium stearate was purchased from Alfa Aesar (Tewksbury, MA, USA). All other reagents and chemicals used were of analytical grade.

### 2.2. Thermogravimetric Analysis

A thermogravimetric analyzer (TGA 1-Pyris, PerkinElmer, Inc., Waltham, MA, USA) was used to determine the thermal degradation of the polymers at the temperature employed in HME and 3D printing techniques. The samples were loaded in aluminum pans and heated from 50 to 500 °C at a rate of 20 °C/min. Ultra-purified nitrogen was used as a purge gas at a flow rate of 25 mL/min. The data were collected and analyzed using the PerkinElmer Pyris™ software (Waltham, MA, USA), and the percent mass loss was calculated.

### 2.3. Preparation of the Filaments Using HME

An 11 mm twin-screw co-rotating hot-melt extruder (Process 11 Thermo Fisher Scientific, Waltham, DE, USA) was used to prepare the filaments required for 3D printing of the floating pulsatile tablets. The standard screw configuration with 3 mixing zones was employed at 50 rpm screw speed in this study. After the filaments exited from the extruder die, a conveyor belt was used to straighten and collect the filaments for easy processing into the 3D printer. Two formulations, HPC alone (100% *w/w*) or in combination with EC (99.5 *w/w* % HPC, 0.5% *w/w* EC)) were mixed in a V-shell blender (Maxiblend^®^, Globe Pharma, New Brunswick, NJ, USA) for 15 min and used for fabrication of the filaments. A temperature of 165 °C was set in all the eight heating zones and torque inside the barrel was observed between 4−5 Nm during the extrusion process. After the extrusion process, the filaments obtained were stored in a desiccator to avoid moisture pickup before they were used for 3D printing of the tablets. The filaments stored outside the desiccator absorbed moisture more quickly compared to the ones stored in the desiccator. 

### 2.4. Mechanical Characteristics of Hot-Melt Extruded Filaments

**Figure 1:**
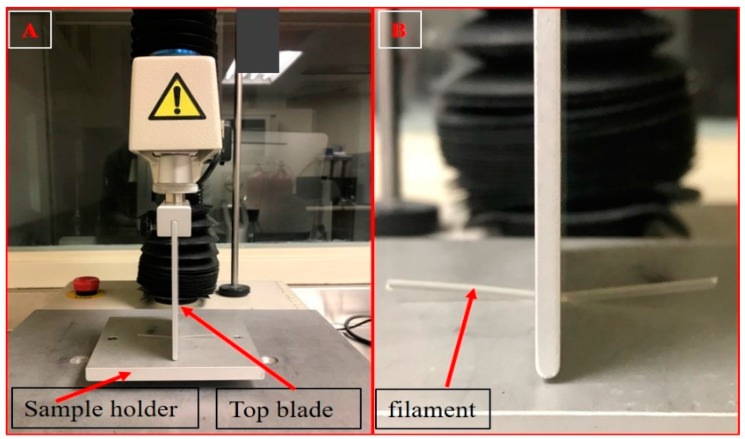
Texture analyzer set up (**A**) and stiffness test of the extruded filaments (**B**).

The mechanical properties of the extruded filaments (flexibility or ductility and brittleness) are the critical parameters that are used to determine the suitability of the filaments for FDM 3D printing (Repka-Zhang test). Texture analyzer (TA-XT2i analyzer and Texture Technologies, Hamilton, MA, USA) were used to evaluate the mechanical properties of the filaments. The filaments were cut into a length of 5 mm and placed on the bottom flat surface of the texture analyzer. The top blade part of the texture analyzer was moved down until it penetrated the filament (0.6 mm) to create a 35% deformation in the shape of the filament. Testing for each single filament formulation was repeated 6 times. The force required to deform the filament was measured and analyzed using Exponent software version 6.1.5.0 (Stable Micro Systems, Godalming, UK). The parameters for the test were as follows: pre-test speed, 10 mm/s; test speed, 5 mm/s; and post-test speed 10 mm/s (Figure 1). The force applied by the feeding gears of the FDM 3D printer on the filament during the feeding process could be co-related with the force applied by the texture analyzer. This provides preliminary data for the assessment of the 3D printability of extruded filaments.

### 2.5. Preparation of the Core Tablets by Direct Compression

Theophylline (57% *w/w*), croscarmellose sodium (8% *w/w*), microcrystalline cellulose (34% *w/w*) and magnesium stearate (1% *w/w*) were sifted using a #30 sieve and blended using V-shell blender (Maxiblend^®^, Globe Pharma, New Brunswick, NJ, USA) for 10 min. The blended physical mixture (175 mg) equivalent to 100 mg of theophylline was compressed into tablets using 8 mm round flat punches by a single punch press (MCTMI, GlobePharma Inc., New Brunswick, NJ, USA).

### 2.6. 3D Printing of Pulsatile Floating Tablets

Initially, the hollow tablets were designed using Autodesk^®^ Tinkercad™ free online software (Autodesk, CA, USA) and saved into 3D printer readable stl. format files. The stl. files were then imported into Ultimaker Cura software (Cura version 4.0, Ultimaker, Geldermalsen, Netherlands). Hot-melt extruded filaments were loaded into an FDM 3D printer (Prusa i3 3D desktop printer, Prusa Research, Prague, Czech Republic), which has an E3D v6 Hot End and a 0.4 mm nozzle and the floating tablets were printed. All the tablets were designed to have the same dimensions (14.5 mm diameter and 7 mm height) but with different shell thicknesses, wall (outer shell) thicknesses, and infill densities (Table 1). The tablets were printed at a nozzle temperature of 190 °C. The other settings used for 3D printing were as follows: bed temperature, 60 °C; nozzle traveling speed, 50 mm/s; layer height, 0.10 mm; and printing speed, 50 mm/s.

**Figure 2:**
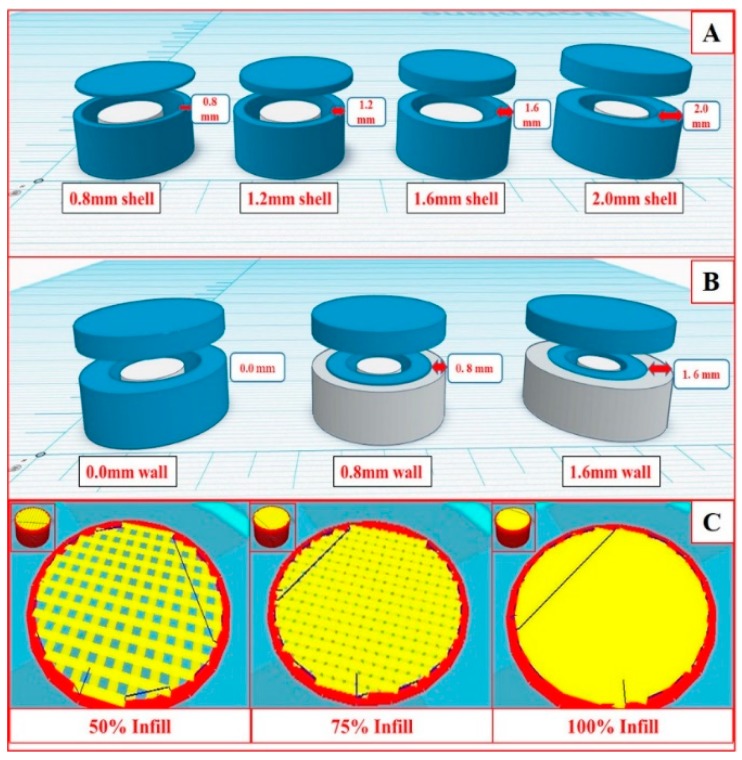
Graphical images of the floating tablets with different shell thickness (**A**), wall thickness (**B**), and infill density (**C**).

The structural parameters of a dosage form that were altered in FDM 3D printing were shell thickness, wall thickness (outer shell) and infill density. The shell can be defined as the total width of the perimeter of the dosage form, whereas the wall was a part of the shell that had different infill patterns and compact structures compared to the inner core. Alteration in the infill density changes the porosity of the structure of the dosage form. The wall has the same composition as that of the shell, but the printing pattern is different from the shell. Firstly, pure HPC filaments were used to print the hollow tablets with four different shell thicknesses (0.8, 1.2, 1.6, and 2.0 mm) as shown in Figure 2A. To assess the lag time, in vitro release studies were conducted for floating tablets of plain HPC and HPC with different concentrations of ethyl cellulose (0.5 to 10 *w/w* %). Secondly, to assess the effect of wall thickness and % infill density of the shell on the drug release profiles, tablets with three different wall thickness (outer shell) (0, 0.8, and 1.6 mm) as shown in (Figure 2B), and three different % infill densities (50, 75, and 100) as shown in (Figure 2C) were printed using the filament containing 0.5% EC. The impact of infill density can only be assessed without a wall. So, a wall thickness of 0 mm was used while printing the tablets with different infill densities.

### 2.7. Loading Capacity and Buoyancy of the 3D Printed Floating Tablets

The printed tablets should remain buoyant in the gastric fluid in the stomach until the pulse release of the drugs occurs. Theoretically, an object can float when the buoyancy force exerted by the fluid is more than the opposite force by gravity (Archimedes’ principle), i.e., if the total force acting vertically on the object is positive. To attain this, the total density of the dosage form should be less than the density of the gastric contents (reported as ~1.004 g/mL) [1]. Based on this principle, the maximum amount of the drug that can be loaded in the proposed floating system and can remain buoyant in the gastric fluid was calculated.

### 2.8. In Vitro Floating Study and Refloating Ability

The in vitro floating study of the 3D printed floating tablets was performed using a United States Pharmacopeia (USP II) dissolution test apparatus (Hanson SR8-plus™; Hanson Research, Chatsworth, CA, USA). The media was 900 mL of 0.1 N HCl maintained at 37 ± 0.5 °C and the paddle speed was set at 50 rpm. To determine the re-floating ability of the printed tablets, tablets were immersed in the dissolution medium for 5 s per hour using a glass rod during the floating study. The test was performed in triplicate. To obtain clear images of the floating tablets, the tablets were transferred to 500 mL glass beakers filled with the dissolution media and the photographs were captured at 0, 2, 4, and 6 h [29,30].

### 2.9. Scanning Electron Microscopy (SEM)

The surface morphology of the extruded filaments and 3D printed floating tablets were studied with a JOEL JSM 5610LV scanning electron microscope (SEM) (JOEL, Peabody, MA, USA) with an accelerating voltage of 5 kV. All the samples were placed on the SEM stubs and adhered by using double-adhesive tape. The samples were sputter-coated with gold under an argon atmosphere using a Hummer 6.2 Sputter Coater (Ladd Research Industries, Williston, VT, USA) prior to imaging.

### 2.10. In Vitro Drug Release Study

The drug release characteristics of the 3D printed floating tablets were determined using a United States Pharmacopeia (USP II) dissolution test apparatus (Hanson SR8-plus™; Hanson Research, Chatsworth, CA, USA). The dissolution media was 900 mL of 0.1 N HCl maintained at 37 ± 0.5 °C, and the paddle speed was set at 50 rpm (29). The samples were collected at the pre-determined time intervals and analyzed for drug content using a UV/VIS spectrophotometer (GENESYS 180, Thermo Scientific) at a wavelength of 272 nm. The calibration curve of *y* = 0.0556*x* + 0.008 was acquired with an r^2^ value of 0.9996. Each test was carried out in triplicate and the collected data were plotted as percentage cumulative drug release versus time.

### 2.11. Statistical Analysis

Statistical analysis was performed by one-way analysis of variance (ANOVA) with Student-Newman-Keuls post-hoc testing using GraphPad Prism 5 software (GraphPad Software, San Diego, CA, USA) with *p* ≤ 0.05 as the level of significance.

## 3. Results and Discussion

### 3.1. Thermal Analysis of the Polymers

The thermal degradation behaviors of HPC and EC are analyzed using thermogravimetric analyzer. From the results and literature reports, it was observed that the percentage loss in the mass was <1% at the temperature (190° C) used for 3D printing of the tablets [31]. A significant mass loss of the polymers was observed at temperatures >250 °C, implying that the polymers were stable during the HME and 3D printing processes.

### 3.2. Filament Preparation and Characterization

Different pharmaceutical-grade polymers were recently investigated for FDM 3D printing of the tablets for various applications. From the available pharmaceutical-grade polymers suitable for FDM 3D printing [27,29], HPC was utilized for the preparation of domperidone sustained release intragastric floating tablets. In this study, HPC was chosen for fabrication of the filaments required as feedstock materials for the development of 3D printed floating pulsatile tablets. In the preliminary studies, EC was added at different concentrations (10%, 5%, 2.5%, and 1%) to HPC to prolong the lag time for the pulsatile drug release characteristics. After all the EC concentrations were studied, the lag time observed was >8 h. The developed tablets with 0.5% EC exhibited a desired lag time of 6 h.

During the HME process, the filaments exiting the extruder die were coiled in irregular shapes and were difficult to process through the 3D printer. Therefore, a conveyor belt was used to straighten the filaments for easy loading into the 3D printer. After solidification, the filaments were coiled and stored in a desiccator to prevent moisture uptake. The filaments that absorbed moisture became soft and squeezed between the feeding gears of the 3D printer. This is because, after moisture absorption, the flexibility of the filaments increased, preventing the melted material from pushing through the heater. Further, the inflow of materials from the printer nozzle was irregular.

After fabrication of the filaments using the HME technique, the mechanical properties of these extruded filaments were tested using a texture analyzer and compared with those of the commercially available polylactic acid (PLA) filaments, which have optimum mechanical properties suitable for FDM 3D printing. For a filament to be considered suitable for FDM 3D printing, it should not either break or be curved aside by the feeding gears during the feeding process because this might result in an inadequate flow of materials through the nozzle. If the filament is too brittle it may get broken by the gears and if it is too flexible it may curve away from the feeding gears because the materials cannot be pushed through the narrow 0.4 mm nozzle of the 3D printer. For this reason, there should be a balance between the brittleness and the flexibility of the filaments.

**Figure 3:**
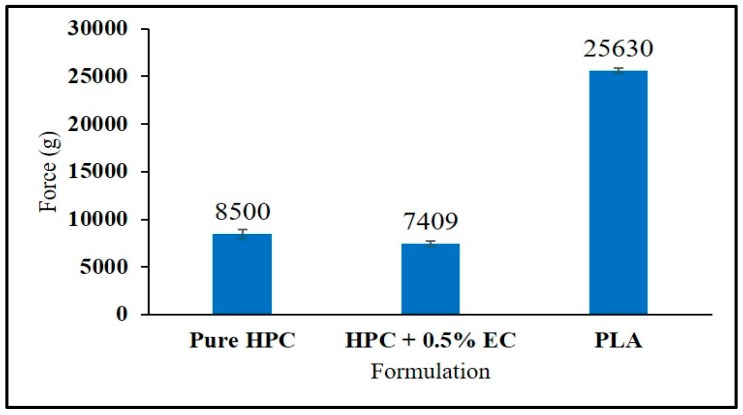
Force values of stiffness test of the hot-melt extruded filaments (error bars represent mean ± S.D).

Even though PLA is considered as a reference material for comparing the mechanical properties of the filaments in 3D printing, it is interesting to note that filaments with a lower stiffness (breaking stress and force) than the PLA filament were good enough for the FDM 3D printing process. This may be due to a variation in the force applied by the gears of the different 3D printers during feeding of the filaments into the 3D printer. In this study (Figure 3), the force required to create a 35% deformation in the shape of the filament was considered as the maximum force that the filaments could withhold during the feeding process without any breaking or squeezing phenomenon in the feeder. Pure HPC and HPC + EC require a lower force of 8500 g and 7409 g, respectively, as compared to that of the reference material PLA (25,630 g). However, these filaments did not show any breaking or squeezing problems during the printing process, which resulted in the fabrication of high-quality floating tablets.

In a study conducted by Chai et al. [29], the authors developed intragastric sustained release floating tablets of domperidone using HPC EXF as matrix polymer for the fabrication of filaments. The authors used 10% (*w/w*) domperidone in the filament composition. The fabricated filaments exhibited the desired mechanical properties suitable for FDM 3D printing. Similar results were observed in the current study. In the earlier study, filaments fabricated using HPC EF and HPC HF grades with 30% paracetamol were not suitable for FDM 3D printing [32]. The filaments produced using both HPC EF and HPC HF were too soft. This may be due to the relatively high drug load of 30% (*w/w*) paracetamol, which acted as a plasticizer and softened the filaments. These studies indicate the significance of drug loading and the properties of drugs in the fabrication of filaments to be utilized in FDM 3D printing.

### 3.3. Physical Properties of the Compressed Tablets

After performing preliminary experiments with different ratios of theophylline and other ingredients, the composition (theophylline (57% *w/w*), croscarmellose sodium (8% *w/w*), microcrystalline cellulose (34% *w/w*), and magnesium stearate (1% *w/w*)) was selected to prepare immediate-release theophylline tablets. All the compressed theophylline tablets demonstrated acceptable uniformity in weight (175.09 ± 5.76 mg), thickness (2.90 ± 0.16 mm), and hardness (4.27 ± 0.70 kp). The disintegration time was <1 min and the tablets showed 100% drug release within 30 min.

### 3.4. 3D Printing of the Floating Tablets

**Figure 4:**
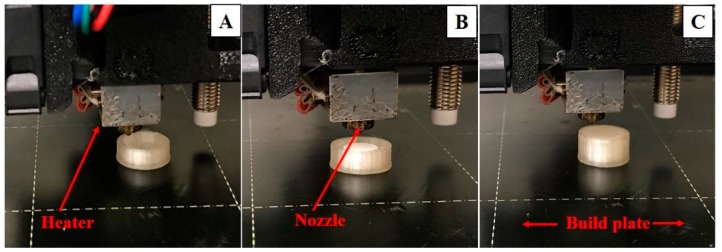
Eighty percent of the printed empty shell of a floating tablet (**A**), placement of a compressed tablet in the shell (**B**), and a completely sealed floating tablet (**C**).

The physical properties of all the printed floating tablets are enumerated in Table 1. 3D printing of the floating pulsatile tablets was achieved in three steps (Figure 4). In the first step, 80% of the tablet shells were printed and the printer was paused. In the second step, the directly compressed core tablets were placed in the 80% printed shell and the final printing was resumed to form a completely sealed floating tablet. This process resulted in the printing of tablets without any structural defects. The thicknesses of the top and bottom were the same as the shell thickness of all the prepared tablets. The tablets printed without any wall had rough surfaces and structural defects in some of the tablets, whereas the surfaces of the tablets with walls were smooth and no structural deformities were observed during the printing process.

### 3.5. Loading Capacity and Buoyancy of the 3D Floating Tablets

For a tablet to remain buoyant in the gastric fluid [1],

F_Buoyancy_ ≥ F_Gravity_. (1)

Equation (1) above can also be represented as follows:

ρL·V_max_ g ≥ (m_s_ + m_t_) g (2)

where ρL is the density of gastric fluid, V_max_ is the maximum volume of liquid displaced (i.e., the volume of floating pulsatile tablet, V_t_), g is the acceleration due to gravity, m_s_ is the mass of shell, and m_t_ is the mass of the compressed tablet. Equation (2) is simplified as Equation (3):

ρL·V_t_ ≥ (m_s_ + m_t_), (3)

where ρL is the density of the gastric fluid i.e., 1 g/cm^3^, and mass of shell (m_s_) + mass of compressed tablet (m_t_) is the total mass of the floating tablet (M_t_). So, Equation (3) was further simplified as Equation (4) as follows:

(ρL·V_t_) − m_s_ ≥ m_t_. (4)

The volume of the floating tablet (V_t_) can be calculated from its dimensions. The optimized floating tablet shell had a weight of 913 mg and a volume of 1155 mm^3^. So, the maximum weight of the compressed core tablet that can be accommodated was 242 mg. In our study, we used a compressed core tablet with a total weight of 175 mg, which is equivalent to 100 mg of theophylline. By optimizing the composition of the core compressed tablets for an immediate-release profile, any drug that is a suitable candidate for a floating pulsatile release system can be delivered with this proposed pulsatile floating tablet.

### 3.6. Floating and Refloating Abilities of the Printed Tablets

**Figure 5:**
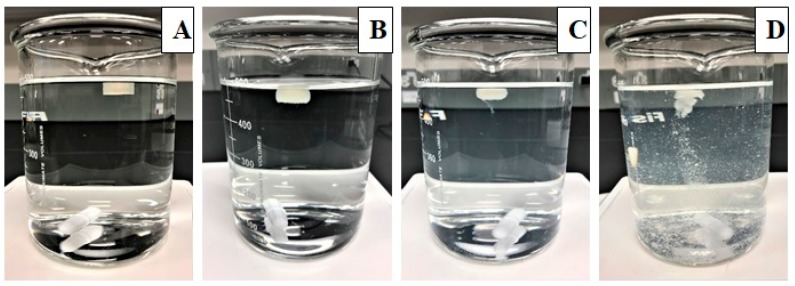
Images of the floating tablets taken at different time points during the dissolution study in 0.1 N HCl: 0 h (**A**), 2 h (**B**), 4 h (**C**), and 6 h (**D**).

All the tablets placed in the dissolution media floated immediately without any lag time. The floating ability of the printed tablets was highly correlated with their density as reported in the previous literature [28]. Tablets with a density of >1 mg/mm^3^ sunk to the bottom of the dissolution vessels. The density of the printed tablets ranged from 0.548−0.941 mg/mm^3^. Tablets with high shell thickness and high infill had higher densities. No difference in the density was observed between the tablets with various wall thicknesses with 100% infill density (*p* < 0.005). When the tablets were immersed in the dissolution media to see their refloating ability, they rose to the top of the dissolution vessel immediately without any lag time. All the tablets showed good refloating ability without any loss of structural integrity. The images of the floating tablets obtained at different time points during the dissolution study are shown in Figure 5.

### 3.7. Surface Morphology

**Figure 6:**
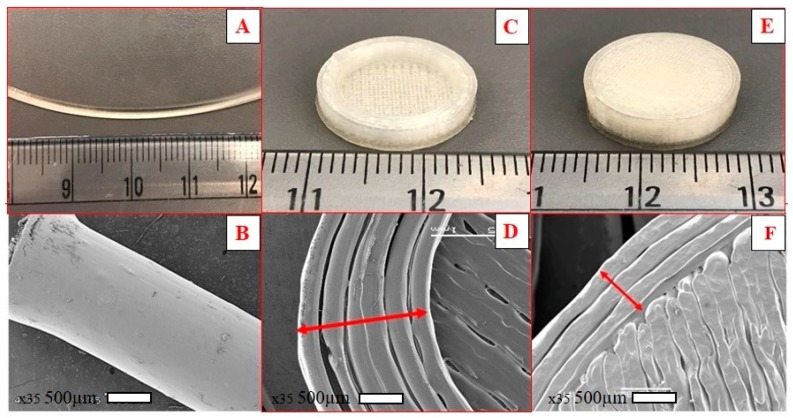
Digital images and representative SEM images of the hot-melt extrusion (HME) filament (**A**,**B**), the cross-sectional structure of the floating tablet showing shell (**C**,**D**), and surface morphology of a 100% infill 3D printed tablet (**E**,**F**).

The surface morphology of the extruded filaments, cross-sectional structure, and surface of the floating tablets are shown in Figure 6. The surface of the extruded filament was smooth and homogeneous without any deformities, suggesting suitability for 3D printing. Filaments that have rough or irregular surfaces will not feed smoothly into the 3D printer and result in irregular shaped tablets. The cross-sectional structure of the floating tablets showed multiple single layers printed side by side to form the shell. The surface of the 100% infill 3D printed tablets showed close adjacent layers without any gaps.

### 3.8. In Vitro Dissolution Study

**Figure 7:**
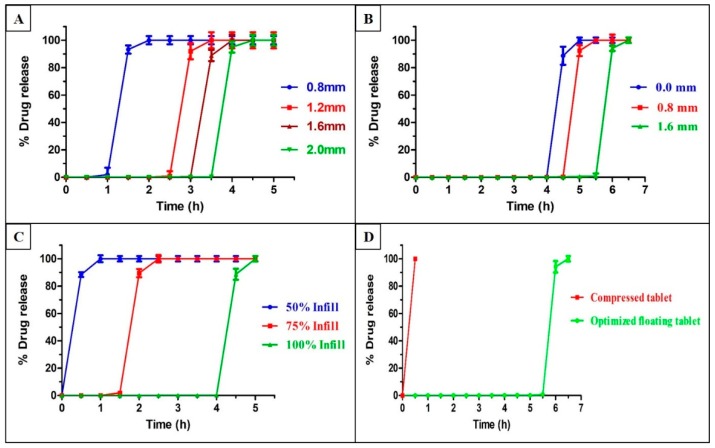
In vitro release profiles of the floating tablets with different shell thicknesses in 0.1 N HCl (**A**), different wall thicknesses (**B**), different fill densities (**C**), and optimized floating tablets and compressed tablets (**D**). Error bars represent mean ± S.D.

The dissolution behavior of the 3D printed floating tablets is shown in Figure 7. From the dissolution data, it was observed that both the tablet geometry and addition of EC into HPC had a significant effect on the drug release profiles (*p* < 0.05). The tablets with higher shell thicknesses had integrity for a longer period of time before the pulse release of API into the dissolution media as compared to tablets with a lower thickness (Figure 7A). The tablets with a shell thickness of 2.0 mm (highest) demonstrated pulse release at the end of 4 ± 0.12 h, whereas those with a shell thickness of 0.8 mm (lowest) showed drug release in 1.5 ± 0.21 h. The tablets with a shell thickness of 1.2 mm and 1.6 mm showed a lag time of 3 ± 0.16 and 3.5 ± 0.08 h, respectively, before complete drug release. Similar results have been reported in the literature on the effects of shell thickness. In a previous study conducted by Maroni et al., the authors printed two-compartment capsular devices, each compartment with different thicknesses and reported that the duration of the lag phase for the pulse release of drugs increased proportionally with an increase in the compartment thickness [33]. This phenomenon can be caused by a higher shell thickness that prevents the entry of the dissolution media into the core for a longer time. The addition of EC to the formulation increased the threshold time for complete drug release (Figure 7B). The tablets with 2.0 mm shell thickness, which had an EC of 0.5%, showed pulse release at the end of 5 ± 0.13 h.

To assess the effect of the wall thickness and infill density of shell on the pulse release of drug, tablets with three different wall thicknesses (0.0, 0.8, and 1.6 mm) and three infill densities were studied (50%, 75%, and 100%). The tablets with higher wall thicknesses prevented the entry of the dissolution media more effectively than those with low wall thicknesses. This is attributed to the closed, compact structure of the wall. This observation was consistent with the results of a previous study [34]. The tablets with 100% infill densities demonstrated pulse release at the end of 4.5 ± 0.26 h, followed by 75% infill (2 ± 0.22 h), and 50% infill (0.5 ± 0.19) (Figure 7C). The higher porosity of lower infill density has a higher surface area and allows easy entry of the dissolution media into the core, resulted in a faster release of API. Yang et al. [35] and Chen et al. [36], who used FDM 3D printing for the development of controlled release dosage forms, reported that a higher fill density caused a reduction in the total surface area and resulted in slower drug release from the dosage forms.

All the prepared floating tablets exhibited a pulse release of drug with different threshold times varying from 30 min to 6 h. The tablets with a specific geometrical structure (2 mm shell, 1.6 mm wall, 100% infill) with 0.5% EC (Figure 7D) were considered optimized formulations for pulsatile release of drugs after 6 h for effective chronotherapeutic treatment of asthma.

## 4. Conclusions

Floating pulsatile tablets with the desired lag time for pulse release of theophylline were successfully developed with the proposed HME coupled 3D printing technique. The geometrical properties of the tablets (shell and wall thickness and infill density) and EC showed significant (*p* < 0.05) effect on the lag time. Thus, the lag time can be varied from 30 min to 6 h based on the requirements. The proposed floating pulsatile system showed high potential to deliver drugs that need high residence time in the stomach and the pulsatile release of theophylline. This strategy reduces unwanted adverse effects and improves patient compliance. In addition, thermolabile drugs can be delivered through this system as the inner core tablet is not exposed to high temperatures involved in the FDM 3D printing process. In conclusion, HME coupled 3D printing is a novel technique to develop low-cost customized drug dosage forms for personalized pharmacotherapy. However, dosage forms fabricated using FDM 3D printing technology need to overcome the challenges in terms of regulatory concerns. Therefore, these floating systems need to be further assessed in in vivo conditions to demonstrate their suitability for patient use.

## Figures and Tables

**Table 1:** Physical properties of the 3D printed floating tablets.

**Filament 1 (100% (*w/w*) HPC)**					
**Shell Thickness (mm)**	**Wall Thickness** **(mm)**	**Infill Density** **(%)**	**Weight** **(mg)**	**Density** **(mg/mm^3^)**	**Floating Duration** **(h)**
0.8 mm	0.8 mm	100	633.13 ± 11.20	0.55 ± 0.01	1.50 ± 0.21
1.2 mm	0.8 mm	100	774.67 ± 12.06	0.67 ± 0.01	3.00 ± 0.16
1.6 mm	0.8 mm	100	868.67 ± 18.72	0.75 ± 0.02	3.50 ± 0.08
2.0 mm	0.8 mm	100	1088.66 ± 7.37	0.94 ± 0.01	4.00 ± 0.12
**Filament 2 (0.5%EC (*w/w*), 99.5% HPC)**					
**Shell Thickness (mm)**	**Wall Thickness** **(mm)**	**Infill Density** **(%)**	**Weight** **(mg)**	**Density** **(mg/mm^3^)**	**Floating Duration** **(h)**
2.0 mm	1.6 mm	100	1086.33 ± 6.66	0.94 ± 0.01	6.00 ± 0.09
	0.8 mm	100	1080.67 ± 10.69	0.93 ± 0.01	5.00 ± 0.13
	0.0 mm	100	1080.00 ± 16.82	0.93 ± 0.01	4.50 ± 0.26
		75	902.33 ± 8.39	0.78 ± 0.01	2.00 ± 0.22
		50	648.67 ± 9.87	0.56 ± 0.01	0.50 ± 0.19
